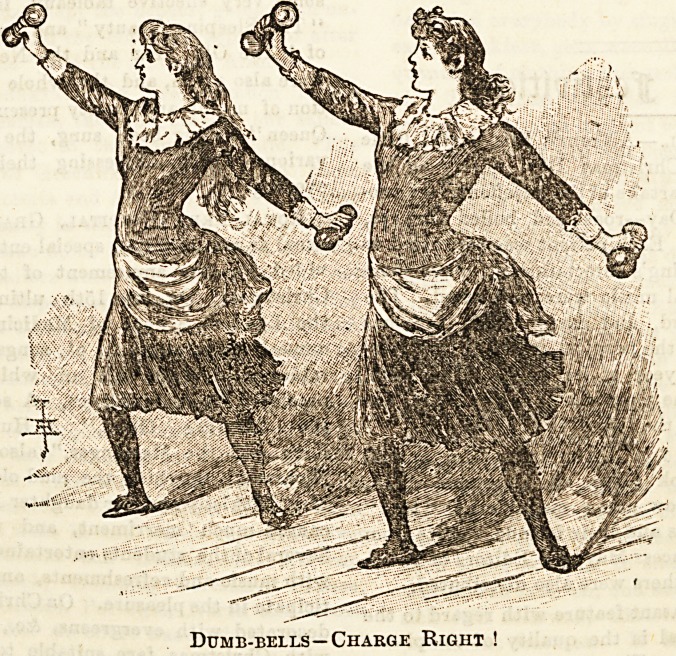# The Hospital Nursing Supplement

**Published:** 1893-01-07

**Authors:** 


"The Hospital" Nursing Mirror.

Being the Extra Nursing Supplement of "The Hospital" Newspaper.

[Contributions for this Supplement should be addressed to the Editor, The Hospital, 140, Strand, London, W.C., and should have the word "Nursing" plainly written in left-hand top corner of the envelope.]

### News from the Nursing World.

### THE QUEEN'S LETTER.

If we attempt to realise the enormous correspondence, which is one of the penalties of Royalty, we can then better understand that an epistle, which is known by high and low, by rich and poor, merely as "The Queen's Letter," must indeed be one of some special interest. Her Most Gracious Majesty addressed to the nation, in her own handwriting, a kindly and affectionate response to the universal tribute of sympathy tendered to her by her people on the unexpected death of her beloved grandson, the young Duke of Clarence. A facsimile of the letter was shortly reproduced, and has been a valued treasure in many an English household during the months which have passed since its issue. On Christmas Day, with Her Majesty's permission, over 38,000 copies of this same letter were distributed to patients in hospitals throughout the kingdom. The seasonable addition of a Christmas card accompanied each copy.

### PRINCESS LOUISE—PRESIDENT.

A first appeal for public support has been recently made by the Aberdeen Association for Nursing the Sick Poor in their own Homes. The Association is a branch of "The Queen Victoria Jubilee Institute," and, as our readers know, H.R.H. Princess Louise became President of the Scotch branch of the Institute on the death of the late Lady Rosebery, who had worked so bravely to get the work successfully started. It has grown and prospered enormously, and over half the "Queen's nurses" trained by "The Institute" are natives of Scotland.

### MALE VOICE CLUB.

Their Royal Highnesses the Princess Christian and Princess Louise, Marchioness of Lorne, have graciously consented to be patronesses of the concert given by the London Male Voice Club (Männergesangverein) in aid of the funds of the Grosvenor Hospital for Women and Children, which is to be held on February 6th, 1893, at the Town Hall, Westminster.

### SMALL PATIENTS AT PADDINGTON.

On hospital fête-days visitors gravitate naturally towards the children's wards, partly, no doubt, from the unconscious attraction which the helpless occupants exercise over the strong and the healthy. Strangers may feel awkward in the presence of adult patients, failing to see that by them a cheery word is always welcomed if offered unaffectedly and not patronisingly. However, the children are always a safe resource, and can be inquired about and their diseases freely, named with no fear of wounded feelings. At St. Mary's Hospital, on the occasion of the laying of the Clarence memorial stone, the little people in the wards received their usual measure of attention, and very dainty the frail little creatures looked in their snowy cots, the pure white curtains relieved by a delicate dash of colour in the shape of rose pink ribbons which had a very pretty effect. The cots were not all full, but the face of each child present proved its claim to the title of patient.

### A FRIEND'S PICTURE.

At various times nurses have asked where they could supply themselves with a photograph of the founder of the Royal National Pension Fund. In No. 47 of "Our Celebrities" an excellent portrait by Walery, of Regent Street, was reproduced, and the publication in question can be obtained from Sampson Low and Marston, Fetter Lane, Fleet Street, for half-a-crown.

### MILL ROAD INFIRMARY.

On January 9th the Guardians will open the first block of the new buildings designed for the Mill Road Infirmary, Liverpool. This block will accommodate 200 male patients, and by the end of March it is hoped that another portion will be fit for occupation. One half of the Nurses' Home will be ready for use on January 9th, and the rooms are well furnished, cosy, and of reasonable dimensions. Each member of the nursing staff will, of course, have a separate bed-room, and there is to be a pleasant sitting-room for the charge nurses and one for the probationers. By-and-bye we hope to give our readers a full description of both Infirmary and Home.

### A POPULAR LECTURER.

Three excellent lectures on practical points connected with home nursing were given recently at Littleborough, Lancashire, by Miss Lucie H. S. Blaydes, better known amongst her audience as "our Sister Lucie." The Women's Liberal Association lent their large hall, which was well filled on each occasion by an appreciative and enthusiastic assembly. The first two addresses were exclusively for women, but by their own urgent desire men also were admitted to the last lecture of the course. The lady lecturer having worked amongst the people as a nurse for some time past has an intimate and valuable knowledge of their special needs, therefore her teaching carries practical weight. Sister Lucie has also given a lecture on nursing to the members of the Girls' Friendly Society, and has promised them another early in February, to which they are eagerly looking forward. Much regret has been expressed at the course of lectures not being more extended, as they were considered far too short by the audience. A more gratifying voluntary tribute to a speaker it would be difficult indeed to imagine.

### A NEW DISTRICT NURSE.

The supporters of the hospital at Weston-super-Mare are anxious to secure the services of a trained district nurse for the sick poor of their town. They have issued a circular on the subject, which sets forth their wishes in a clear and business-like manner. Such an excellent scheme should certainly receive the support which it deserves, and doubtless the £80 per annum which will cover all expenses, will promptly be guaranteed by the friends who are interested in the movement.

### THE LATE MISS STAINS.

The Liverpool Royal Infirmary, and the Training School and Home for Nurses attached to it, have sustained a heavy loss in the death of Miss Stains, who, for over eleven years has occupied the responsible position of Lady Superintendent to both institutions. Miss Stains's health gave much ground for anxiety in the autumn of 1891, and she had to give up work for several months, returning much benefited by the quiet and rest she had enjoyed. It was not until September last that her health again gave way, and she was confined to her room until her death, which occurred on Thursday night, December 29th. Miss Stains had had a long and varied experience, having commenced her career in 1867, at the Herbert Hospital, Woolwich, whence in 1871 she entered for training as a Nightingale probationer at St. Thomas's Hospital, becoming in due course a Ward Sister, and subsequently Matron of Wolverhampton Hospital, from which place in 1881 she entered on the duties of Lady Superintendent in the institution in Liverpool. During the erection of the New Royal Infirmary in Liverpool, her experience and advice were invaluable, and her energy and constant supervision of all the essential details were very conspicuous. It is impossible to speak too highly of the thorough manner in which her very onerous duties were discharged, the interests under her care being her first thought, and her extensive knowledge of a nurse's training in its many aspects, and of hospital management, contributed alike to the efficiency of the staff and the working of the hospital. Whilst strict and decided, her aim was to be just, and the goodwill of all under her is evidence of the estimation in which she was held. The Committees of both institutions feel they have lost a most valuable officer, and her death is a matter of general regret. In fact, speaking practically, it may be said that all that is good, both structurally and administratively, in the Infirmary owes its origin to Miss Stains' great abilities, and the people of Liverpool are the first to admit it. The account of the new buildings of the Liverpool Royal Infirmary which appeared in The Hospital of October 10th, 1891, made special mention of the "great practical thoroughness of Miss Stains, the Lady Superintendent," and the value of her influence on the plans is shown by the disastrous failure made of the servants' cubicles, about which, through an oversight, she was not unfortunately, consulted.

### A LADY DOCTOR AS A LECTURER.

Some lectures on sick nursing were recently given at Tranent by Dr. Elsie Inglis, in connection with the Y.M.C.A. Dr. Edmund Price, of the St. Andrews Ambulance Association, Edinburgh, held a pass examination afterwards, and twenty-six candidates received certificates. Dr. Price expressed himself much pleased with the practical work, and also with the oral examinations. Dr. Elsie Inglis has lately been appointed House Surgeon to the New Hospital for Women in Euston Road, London.

### A WISE MEMORIAL.

A meeting has been held at Alnwick by the subscribers to the memorial to the late Duchess of Northumberland, and it has been decided to devote the interest of the money raised to a most useful object. The subscriptions are to be invested as a memorial fund, and half the dividends and interest received will be paid to the acting secretary of the Alnwick Nursing Fund, to provide nursing requisites in connection with this fund; the other half will be placed in the hands of a committee of ladies, to be used for the training of village nurses, and in "providing sick-room requisites in the neighbourhood of Alnwick." It would be difficult to imagine a better or more lasting "memorial."

### ONE WARM GARMENT.

We often have to note pleasant facts in connection with the Glasgow Sick Poor Nursing Association, and now we must cordially sympathise with the kindly appeal made by two of its workers. In the *North British Daily Mail* a letter has appeared asking for the help which will assuredly be freely bestowed by the healthy and prosperous, from whom it is requested—a warm garment and a toy for each of the sixty poor children whom the district nurses are tending just now in their own often dreary homes. The two gifts will please parents and patients in an almost equal degree, and we have every hope of hearing that the modest demand has received an adequate response. In that case the New Year's pilgrimage of each enthusiastic nurse will have taken something of the character of a festive and seasonable kind of progress.

### PRACTICAL PROGRESS.

A meeting convened by Provost Anderson was held at Anstruther, on December 16th, to discuss the formation of an Association for providing a trained nurse for the sick-poor in the districts of East and West Anstruther, Cellardyke, and Kilrenny. Letters were read from several persons who were prevented from attending, and these all expressed much sympathy with the movement, and promised pecuniary aid. All the doctors present agreed as to the urgent need for a permanent nurse, and much kindly interest in the subject was evinced. A fair proportion of the sum needed for the purpose has already been promised, and there seems little reason to doubt that the £70 or £80 per annum, which is required, will be forthcoming with but little delay. The scheme seems likely to be well-organised and also well-officered, for it is in the hands of an influential committee. The Q.V.J.I. in Edinburgh has been communicated with, and definite arrangements will doubtless shortly be completed.

### OUR AMERICAN SISTERS.

Our American contemporary, *The Trained Nurse*, has some very good articles in the December number. "Memories of Bellevue," by Miss Robins, contains some charming little scenes from every-day life in the wards. An account of one incident which occured during the fire which destroyed the old pavillion is so specially good, we must quote it for the benefit of those readers to whom it is new: "One old woman, supposed unable to move, was found two flights down, and at the extreme other end of the hospital. Full of dismay at seeing her there, the nurses of the nearest ward carried her in and laid her on a bed. 'You will please to remember,' she remarked sharply, as they gently took off the blanket which she had wrapped round her 'that this blanket belongs up in twenty (ward) and not down here.'" Any one accustomed to hospital patients knows how carefully many of them guard the interests of their own particular wards, often even making it their business to report borrowing or removing which has taken place during their nurse's temporary absence. But at a moment of such deadly fright as the presence of fire occasions, there is something pathetically conscientious in the faithful old woman's remarks.

### The Development of Children by Gymnastics.

III.—DUMB-BELL EXERCISES.

THE next set of exercises to be noticed is that in which dumb-bells are used. These are, perhaps, the best known and most practiced, as the bells are portable, and at the same time most effective in the result of their use.

There is a great difference of opinion as to the suitable weight of the bells. Some maintain that they should be heavy, so that the muscles may be well developed by their use, while others declare that they ought to be as light as possible, and merely serve as something to hold, since it becomes more interesting to the performer of the exercise. Now in this, as in most matters, it is well to strike the happy medium. If the dumb-bells are very heavy, the effort of lifting them becomes so great that by the time an exercise has been repeated once or twice the pupil is tired, and the chances are, the rest of the exercises are slurred over, or the muscles strained. Even if the weight is not quite so great as to cause this result, a heavy dumb-bell must of necessity produce laboured movement, and though the muscles are duly developed, the action becomes laborious and the movement ponderous. In our endeavours to train the physical powers of the young, we must bear in mind that strength without grace and agility, becomes almost a deformity. We hardly wish our children to develop into nineteenth century Samsons, neither would mothers be pleased to think that they might be destined to chaperon female Sandows to the dances of the future. It is evident then that for a satisfactory result, agility and grace must be combined with strength, and by using a sufficiently heavy dumb-bell to insure a certain resistance to the muscles whilst exercising, and yet one light enough not to impede free movement, we shall secure the desired end, and turn the natural vivacity of the child into a channel where, rightly guided, it may result in a mature combination of health, energy, and beauty of form.

DUMB-BELLS—CHARGE RIGHT!

We sometimes hear people ask how it is that disease is more prevalent in our times than it was with the ancients. May it not, perhaps, be accounted for by the fact that in olden days the men made it one of their chief objects in life to harden themselves by all manner of exercise and outdoor occupations? Wrestling, running, leaping, rowing, swimming—all these tend to set in full activity the circulation, a very important factor in the consideration of the health of the body. The blood is the natural purifier of the system, and must therefore be far better than artificial devices; medicine is all very well, but as a last resource when Nature, fails to right herself. Writing on the subject of health, Fuller very quaintly says: "The use of exercise does conduce very much to the preservation of health . . . is scarce disputed by any; but that it should prove curative in some particular distempers, and that, too, when scarce anything else will prevail, seems to obtain little credit with most people, who, though they will give a physician a hearing, when he recommends the frequent use of riding, or any other sort of exercise, yet at the same time look upon it as a forlorn method, and the effects rather of his inability to relieve them han of his belief that there is any great matter in what he advises. Thus by a negligent diffidence they deceive themselves, and let slip the golden opportunities of recovering, by a diligent struggle, what could not be procured by the use of medicine alone." (Fuller's "Medicina Gymnastica."

The function of the blood is to supply warmth and new life in the form of fresh particles to those parts of the body which by the exertions attending every slightest movement, have been rendered "waste" or worthless, and changing the "new for old," it carries with it the useless matter which would otherwise breed all manner of ills, and then returns to the heart to be again purified. Blood, then, is, as Maclaren reminds us, "liquid flesh," and to keep it pure we must do all in our power to promote active circulation, and this is done most effectually by exercise.

To many children it is impossible to supply ample space or opportunity for some of the best and pleasantest of these, as rowing or swimming; neither is it always possible or advisable to devote one's whole recreation to leaping or wrestling, especially in the case of girls, so again we fall back on the varied resources of the gymnasium and advise their free use.

Turning our attention at present to the dumb-bells, let us take some of the chief movements. First, then, the bells should be grasped one in each hand with the fingers touching the sides, and the arms allowed to hang straight from the shoulders so that the axis of the bell is horizontal and lies from front to back. The position should be the same as described for the bar-bell — strict "attention," shoulders back, chin slightly raised, eyes front, waist kept well in, knees stiff, heels together, and feet at an angle of 45 deg. The bells are then turned (without raising the arms so as to bring the fingers facing the front, and then back again to the first position. This is repeated several times, and is a capital exercise for strengthening the wrist muscles. It is customary in calisthenic classes to repeat this movement with the arms in different positions—stretched out from the shoulders in front of the body, then on a line with the shoulders and stretched out to either side; lastly, above the head with the arms extended as high as possible. This, however, is not actually necessary, though it helps to give a variety; indeed, it gives an undue proportion of time to the development of one set of muscles.

The next exercise starts from the first and fundamental position; the right arm is kept stiff and raised slowly outwards to a level with the shoulder, then as high as possible above the head, the body being bent from the waist, and the head turned so as to look at the raised bell. The arm is then lowered, and the left raised similarly, and, at the same time, the body bending over to the right. This should be repeated about half-a-dozen times; it seems to strengthen the abdomen muscles, the flexor muscles of the head and neck, and the extensor muscles of the arm.

A very good dumb-bell exercise is the one represented in our illustration. Starting from attention, the bells are raised and placed on the hips; at the words "charge, right," the right arm is extended above the shoulder, and the head thrown well back, eyes turned to the bell, and the left arm in a line with the right, and, therefore, holding the bell below the waist; at the same time the right foot is lifted, and while the left knee is kept stiff, the right is bent and advanced a pace forward to the right. At the word "recover," the heels are again brought together, and the bell placed on the hips. "Charge, left," gives the order for the same exercise reversed. In each case every movement of this exercise must be executed at the one word of command.

There are various other exercises which might be mentioned, but there are also many good books which have illustrated them far better than a limited space will here allow. Among these may be highly recommended to all who are desirous of practising the exercises Professor Hoffmann's very excellent book on "Home Gymnastics," a work deserving the highest attention as a really practical and helpful guide to vigorous health.

### Christmas Festivities.

EALING COTTAGE HOSPITAL.—Although this useful little hospital was nearly full on Christmas Day, nearly all the patients were deemed fit to partake of the excellent fare provided for them on Boxing Day—roast and boiled turkeys, plum pudding and mince pies. Each patient was permitted to invite one friend for the evening's entertainment, when some good songs and instrumental music were performed. The wards were prettily decorated, and many useful presents were received from friends of the hospital, which is now commencing the twenty-second year of its work. Miss Reid, who contributed greatly to the pleasures of the patients, has held the post of matron since the day of the opening.

EAST LONDON HOSPITAL FOR CHILDREN.—The annual Christmas entertainment took place on December 27th, when a Punch and Judy show and a magnificent "tree" were provided for the patients and their friends. Gifts from the Duchess of Teck and Princess May, the Editors of *Truth* and *Little Folks*, and many others were also distributed.

GUY'S HOSPITAL.—One pleasant feature with regard to the entertainments at this hospital is the quality of the pianos which are heard in the wards. They are all good, and they are all kindly lent by eminent makers. The decorations at Christmas were most excellently managed, and the wards vied with each other in beauty. The nigger troupe, "The Guy's Unaccountables," enjoy undiminished popularity.

KING'S COLLEGE HOSPITAL.—The patients had a quiet and happy Christmas Day, and were entertained with a sacred concert. On Boxing Day Christmas fare and other pleasant and seasonable provision was made for both patients and staff.

LONDON HOSPITAL.—The Christmas festivities for the patients at the London Hospital were this year held on Christmas Eve, and the entertainments in the wards were kept up with the usual spirit and animation. The wards were prettily decorated with evergreens, and much ingenuity was shown in the many devices and mottoes which ornamented the walls. The tea in most of the wards began about half-past four, and the tables looked very inviting with their piles of cake, fruit, and crackers. By six o'clock various entertainments were in full swing. In several wards dramatic performances had been got up, followed by magic lantern shows, recitations, and songs. Plenty of help seemed to have been obtained from all quarters, and the sisters of the different wards, upon whose shoulders falls the responsibility of organising, arranging, and providing the evening's amusements, may be most heartily congratulated upon their success. Visitors, as well as patients, cannot fail to have spent a most enjoyable evening, and will own that Christmas in hospital is far from being the dismal affair some people imagine. The Christmas trees for the children, and the annual exhibition of Punch and Judy went off brilliantly on January 3rd. Hundreds of visitors were present, in spite of the bitter weather. Amongst these were specially noticeable matrons from many of the other London hospitals, as well as a large section of doctors and other friends interested in the East End. From basement to attic the whole of this enormous pile of buildings was, as usual, open to inspection, and many and cordial were the commendations heard on all sides.

NEW HOSPITAL FOR WOMEN.—The Christmas entertainment at the New Hospital for Women, Euston Road, was given on Boxing Day. In the afternoon the patients, after a dinner of turkey and other Christmas fare, received their friends. At six o'clock a representation of Mrs. Jarley's Waxworks was given by the sisters and nurses, followed by some very effective tableaux, including four scenes from "The Sleeping Beauty" and an exceedingly pretty picture of "The Old Year and the New." A few character songs were also given, and the whole terminated with a distribution of useful and pretty presents to all. "God Save the Queen" having been sung, the patients retired to their various wards, expressing their great enjoyment of the evening.

ROYAL FREE HOSPITAL, GRAY'S INN ROAD, W.C.—As usual at this hospital, special entertainments have been provided for the amusement of the patients and nurses at Christmas. On the 15th ultimo the lady students of the London School of Medicine for Women gave their annual entertainment of songs, dramatic sketches, and tableaux vivants, all of which were very highly appreciated by the audience. A scene from "Alice Through the Looking Glass," "Humpty Dumpty," "The King and the Messenger," also a scene from "Nicholas Nickleby," in which the mad old gentleman makes love to Mrs. Nickleby and her daughter Kate over the garden wall, caused much merriment, and were specially applauded. Several of the students entertained the patients in the wards with music and refreshments, and thus all were able to participate in the pleasure. On Christmas Day, the wards being decorated with evergreens, &c., the patients were regaled with Christmas fare suitable to their condition, and on Boxing Day a special tea was provided in each ward for the patients and their friends, the nurses vieing with each other to make the evening pass pleasantly with games, &c. On the 27th ult. an excellent dramatic performance of J. M. Morton's comedy, "Old Honesty," was given in the Marsden Ward by the Rev. C. J. Parker (the chaplain of the hospital) and his friends, and on the 29th the Christmas festivities were concluded by a Christmas tree in the Milne Ward for the juvenile patients, when over 100 children, past and present patients, had tea, and received suitable presents.

ST. BARTHOLOMEW'S.—The wards at St. Bartholomew's Hospital were decorated according to custom, and the patients enjoyed a substantial Christmas dinner on Sunday, December 25th. They also had a regular festival tea, and enjoyed visits from their friends and relations during the afternoon.

ST. THOMAS'S HOSPITAL.—At St. Thomas's Hospital Christmas Day was celebrated on December 27th, when the patients had their usual festival fare, and the probationers afterwards gave their annual carol singing in the wards. The Nightingale probationers' own Christmas dinner is provided, we believe, by Miss Florence Nightingale herself. There is a concert in each of the wards during the octave of Christmas, on whichever evening proves most convenient for the nursing staff, and also for those friends who so kindly contribute by their talents to the entertainment of the patients. The fine wards at St. Thomas's Hospital lend themselves very suitably to those decorations which do so much credit to the ingenuity and patience of sisters, nurses, and students. On New Year's Eve a thoroughly good programme was presented to the patients in the Charity Ward, which was illuminated by many coloured lamps, showing off the decorations to perfection. Miss Cooke, who is always a favourite, gave a very finished rendering of "L'invitation à la Valse." Mr. Lionel Brough gave some of his funny stories, and was loudly "asked for more." Mr. Algernon Newark received a hearty encore for his amusing account of a village penny reading, and treated his hearers to a clever imitation of Beerbohm Tree and other well-known actors. Mrs. Edith Dick sang charmingly, both she and Miss Florence Venning winning sincere applause by their sweet voices and finished performance. Mr. Ben Jones on the banjo, and Mr. Walters in the popular coster song, were both highly appreciated. Mr. Fox Symonds, who had kept the concert going with unflagging energy, sang last, and the company separated after singing "Auld Lang Syne" and the National Anthem.

Seamen's Hospital Society.—We owe so much to the labour and bravery of seamen, that the care of our sailors must always be of interest to the general public. The Dreadnought Hospital at Greenwich contains 235 beds, and the branch in the Victoria and Albert Docks 18, making a total of 253 beds constantly in use. At these two hospitals during the year no less than 3,500 in-patients were treated-while over 10,000 sick sailors were attended to in the out patient departments and dispensaries, one of the latter being in the East India Dock Road and the other at Gravesend. When ill, sailors will travel from all parts of the world for treatment at the Dreadnought, and they come with the full knowledge that on arrival they will be admitted and receive the best medical or surgical treatment. The wards are full of men who may have fallen from aloft in a gale; or have suffered from exposure, dysentery, and the effects of hot or cold climates. Every nation finds a home here, and last year there were in the wards representatives not only from Great Britain and Ireland, but Norway, Sweden, Russia, Denmark, Holland, Germany, France, Spain, Italy, Turkey, Greece, Portugal, North and South America, Africa, India, &c. An effort was made to cheer the sailors on Christmas Day. They were supplied with turkeys and plum pudding; the wards were decorated with evergreens and many quaint mottoes and sayings of the sea. On Boxing Day the men were allowed to have a sing-song and smoke in one of the wards, an entertainment which those who were well enough thoroughly enjoyed. There has been an entertainment for the nurses, and a Christmas tree on the 10th inst. will complete the agreeable Christmas programme.

Sussex County Hospital.—The Christmas decorations at the Sussex County Hospital, Brighton, were of a most elaborate description this season. There was a fine Christmas tree in the children's ward, and over each cot, suspended by a bright red ribbon, hung a stocking filled with suitable gifts, whilst graceful drapery and fairy lamps with flags over the doorways, made a charming display. The other rooms were tastefully adorned, "Father Christmas" attracting much attention in Lady Jane Peel ward, whilst the fairy lamps and a tiny snow scene were greatly admired in Egremont.

West Ham Hospital.—On Tuesday last the usual annual tea and entertainment took place. About seventy persons, who had been in-patients during the past year, sat down to a repast kindly provided by the Chairman, Alderman Hay. Afterwards all adjourned to the grand Christmas tree, from which each received a useful present. As many as possible of the in-patients were moved where they could hear the different items gone through, but those too ill were well looked after in the wards. The entertainment, given by the staff and friends, was excellent, and right well did each do her or his part: in fact, it was the most successful of the many good entertainments yet given at this institution. The tableaux passed off splendidly, particularly the one representing "Custard Without Eggs," copied from Messrs. Birds advertisement. When all did so well it seems invidious to mention any name in particular, but we are compelled to take notice of the acting of Miss Ough and Mr. Thompson in their respective parts. The stage management was in the able hands of the Rev. J. W. Eisdell and Dr. Hillier, and so well did they perform their duties, that not a single hitch' occurred. The stage was kindly lent by Mr. Reed.

Westminster Hospital. — At Westminster Hospital Christmas has been observed with a good deal of spirit this year. Christmas Day having fallen on Sunday rather tended to a prolongation of the customary festivities. On Christmas morning the choir boys belonging to the hospital chapel delighted everybody by singing carols at six o'clock, and after early breakfast, gifts were distributed to the patients, a large quantity of clothing and useful articles being thus disposed of. The ladies who visit in the wards very liberally subscribed £34, to be laid out by the matron at her discretion, and upwards of £10 was spent in the purchase of flannel, a step which may not inaptly be described as an effort in the direction of "preventive medicine." Toys, sweets, cards, and books had been sent by numerous friends, all of which gave the patients a great deal of pleasure; but the Queen's letter to the nation, a facsimile copy of which had been kindly sent by Messrs. Raphael Tuck and Sons to every patient, was, perhaps, the most appreciated, many patients expressing their intention of taking it home and framing it. On Boxing Day the good cheer generally associated with Christmas was forthcoming; the usual diets were entirely suspended, and, subject to the permission of the medical officers, every patient in the hospital was provided with turkey or chicken and sausages, followed by plum pudding; and in the case of the men well enough to take the journey to their convalescent ward, by the pipe which it is such a joy to them to smoke. Tea of a festive character was served later on, and such entertainment as is suitable to sick folk followed, Christmas trees and bran pies being prominent features. It is needless to say that the wards were very tastefully decorated with flowers and evergreens, principally the work of the resident medical staff, who are always forward to undertake any labour that can in any way promote the comfort or pleasure of those under their charge. The sisters and nurses gave themselves up entirely to the entertainment of the patients.

University College Hospital.—The Christmas entertainment at University College Hospital took place on December 29th. The wards were prettily decorated and the children had a magnificent Christmas tree for their own particular enjoyment. Tea was provided at five o'clock and a number of people interested in the hospital visited the institution during the afternoon. A capital concert was given in the out-patient department at half-past six and all patients who could safely be transferred there were allowed to be present. For those too ill or too helpless to be moved, amusement was provided by "The Grosvenor Quartette," who spent the evening in giving an excellent selection of music in the different wards.

We have to thank our readers for numerous contributions respecting Christmas entertainments, presentations, &c. Many of these must be held over till next week in consequence of the great pressure upon our columns.

### Our Christmas Parcels.

To all our readers who so readily gave us such admirable help in sending away parcels at Christmas, we give our heartiest thanks, and we wish we could print all the grateful remarks of the kindliness of HOSPITAL readers which greeted the arrival of the welcome parcels at the various hospitals. Everybody who knows anything at all about it, will admit that the difficulty of providing amusement or gifts for the inmates of our hospitals at Christmas is very considerable, but there are hundreds of workers who could, if they would, take this load at least off the backs of their cheery, hard-worked sisters in the wards. There are many women who cannot work actively among our sick, but who are perfectly able to form a valuable auxiliary staff in such matters as Christmas festivities.

There were not so many entries for the competitions as there were last year, but our bundles generally had increased in size and value very considerably. For the first prize of 5s. for the best pair of socks knitted by a nurse, we decided in favour of Nurse Francis, of the Acland Home, Oxford, whose socks were well made in every point; while the prize of 5s. for the best socks by a HOSPITAL reader was won by Miss Chisnall. Nurse Elizabeth Bishop carried off the prize of 10s. for the best made flannel shirt, and Miss Maria Spencer won the 10s. prize for the best made flannel petticoat. There were only two competitors for the dressing gown award. Nurse Child sent in a grey blue gown, made after the pattern of the prize jacket of 1890, which buttoned up everywhere, and is in every respect excellent for a helpless invalid; and Nurse A. F. Heanley sent in a beautiful grey flannel wrapper, feather-stitched down the front, neck and sleeves, so well cut and made that it was impossible to say which was the better of the two, and we decided that a prize for each was the only way out of a difficulty. Nurse Blain sent a very kind letter and some beautiful underlinen; from Nurse Little we received three lindsey petticoats; from Miss E Bishop, knitted petticoats and a cardigan jacket; from Miss Disney, two pairs of socks; from Miss Ayston, knitted petticoats; from Miss Graham, socks; from Miss Shore, socks; from Mrs. William Black, twelve petticoats, four pairs of socks, and two pairs of mittens. Nurse Talbot sent a very good sort of crossover and some socks; Miss Mary Bishop sent us socks; Nurse Makins three children's knitted petticoats and a comforter; while some excellent underlinen was sent by Miss Heanley, and an anonymous friend gave a cardigan jacket and three pairs of socks; Miss Macrae gave some serge petticoats and some socks; Miss Lockyer some crossovers; some "nameless" friends gave flannel petticoats and mittens, and we had a huge bundle of miscellaneous goods from the Brassey Holiday Home. There were socks from Nurse Smith, Nurse Trump, Nurse Mundy, and a flannel petticoat from Eleanor Williams. All this host of things we sorted and divided, and sent a big bundle to the Children's Hospital, Shadwell, St. Mary's Cottage Hospital, Plaistow; the Great Northern Hospital, the Hospital for Women and Children in the Waterloo Road, Charing Cross Hospital, King's College Hospital, Middlesex and the London Hospital respectively. These were no sooner departed than a very good friend, called "Lina," left a great package of petticoats, socks, stockings, and warm things of every sort, and Madame Monchablon and her staff of type-writers very kindly sent two parcels of prodigious size, containing petticoats, gloves, socks, stockings, comforters, knitted hoods, vests, and last but not least, some of the well-made type-written books similar to those sent us last year. These unexpected additions enabled us to start again, and we gave children's things to Cheyne Hospital for Children, the Home for Incurable Children at Maida Vale, and the rest we divided between Westminster Hospital, the Woolwich Hospital at Shooter's Hill, and an extra bundle of socks went down to the "London." In all, twelve hospitals were helped, and we hope that year by year our readers will carry on this work, which at small cost causes so much rejoicing.

### Everybody's Opinion.

*[Correspondence on all subjects is invited, but we cannot in any way be responsible for the opinions expressed by our correspondents. No communications can be entertained if the name and address of the correspondent is not given, or unless one side of the paper only be written on.]*

### NURSING IN TYPHOID.

A NURSE writes from Cairo: May I be permitted to send a few lines to your worthy paper on the nursing of typhoid fever. Symptoms in hæmorrhage are not always well marked. I can speak from experience, as from time to time for over twelve years have I nursed typhoid fever. Most assuredly, if the temperature falls suddenly to normal, or below, cold sweat or cold extremities, with weak pulse, pallor of countenance, or faintness, look for hæmorrhage; yet the latter may and will occur without any of the previous symptoms. Repeatedly during my nursing career my patients have had hæmorrhage profusely, where temperature has remained from 103 to 105, with quick bounding pulse, flushed face, and bright eyes. I have found cases of hæmorrhage as a rule do well, but I have lost a few where the temperature has risen to 108 and 109. In cases of perforation, invariably one can tell what has taken place from aspect, cold sweat, feeble pulse, profuse diarrhœa, &c. Careful, good nursing is the most essential point in typhoid fever, absolute quiet to be maintained throughout the disease, but more especially so when hæmorrhage occurs, keeping patient on back for 48 hours after the latter has ceased, with ice bag to abdomen, giving as little fluid as possible to drink as long as strength is well maintained. Brand's essence is invaluable, as beef-tea frequently increases action of bowels, and milk is liable to curdle. I have found nutritive essence of Brand's essence 2 oz., milk 1 oz., yolk of egg 1, answer admirably, given every fourth hour for 48 hours, or longer, if hæmorrhage has not ceased. Where skin does not act, sponging the body with tepid water helps to lower the temperature, is refreshing, and often produces sleep, but should only be done by the skilled hand. With proper care no bed sores should ever occur, and mouth ought to be kept perfectly clean. Disinfecting stools and bed linen ought never to be omitted. Regarding medicine, solids, &c., the trained nurse will obey her doctor.

### Obstetrical Society of London.

THE next written examination of midwives will take place at 20, Hanover Square, on January 11th, at eight p.m. The circular, which bids candidates send in their applications before December 28th, is dated December 22nd, which seems very short notice to give them.

### Notes and Queries.

### SPECIAL NOTICE.

The contents of the Editor's Letter-box have now reached such unwieldy proportions that it has become necessary to establish a hard and fast rule regarding Answers to Correspondents. In future, all questions requiring replies will continue to be answered in this column without any fee. If an answer is required by letter, a fee of half-a-crown must be enclosed with the note containing the enquiry. We are always pleased to help our numerous correspondents to the fullest extent, and we can trust them to sympathise in the overwhelming amount of writing which makes the new rules a necessity.

### Queries.

(1) *Washed Bandages.*—Can anyone tell me the best method of washing bandages used by a cancer patient?—*Box, Wilts.*

(2) *Nurses at Chicago.*—Can you give me any information about reduced fares for nurses to the Chicago Exhibition, as I should greatly enjoy a short sea voyage and pleasant change at somebody else's expense?—*E. F.*

(3) *Photograph.*—Where can I get a portrait of the founder of the Pension Fund for Nurses?—*Jane.*

### Answers.

(1) *Washed Bandages (Box, Wilts).*—The washing of bandages in such cases should not be attempted unless there is very urgent need for economising the unbleached calico, which can now be bought so very cheaply. When it has to be done, each bandage should be immersed in strong carbolic lotion ($\frac{1}{20}$), or in perchloride of mercury ($\frac{1}{1000}$), so that disinfection should be perfectly secured before washing and boiling is afterwards accomplished.

(2) *An Enquirer (E. F.).*—We can give you information regarding the actual journey, but should like to know whether you are in a position to meet the heavy expenses of travelling and living in a strange land at a specially costly season. What do you mean by "somebody else"? You must write more clearly.

(3) *Photograph (Jane).*—A good picture can be obtained from Messrs. Sampson Low and Marston Fetter Lane, Fleet Street. It came out in "Our Celebrities," No. 47, price 2s. 6d.

### Forty Years of Nursing.

### THE LATE MRS. WARDROPER AND THE NIGHTINGALE SCHOOL.

As briefly stated in our last issue Mrs. Wardroper has gone to her rest full of years, with a record so honourable as to make her second only to Miss Nightingale on the roll of eminent women to whom modern nursing owes its origin and success. Mrs. Wardroper was appointed matron to St. Thomas's Hospital so long ago as 1854, and she found a state of things existing in the institution at that time which would appall most modern matrons. The sisters of those days were without any training or nursing experience of any kind. They were selected by the treasurer, and belonged mainly to the housekeeper class, in which capacity they had usually exhibited some aptitude for management and economy. The buildings were ill-adapted to hospital purposes, but Mrs. Wardroper proved equal to the emergency, and by sheer hard work and pluck gradually introduced changes which prepared the way for the establishment of the Nightingale School some six years later. Mrs. Wardroper's first reforms were directed to the careful selection of suitable women of proved character to fill the post of nurse, and, by effective supervision over the conduct of the staff, she gradually obtained such control that the wards of St. Thomas's Hospital became among the best administered in the country in those early days. Her next step was to select the sisters from the nurses, so as to secure that every sister should have had practical training in her duties, and so be thoroughly familiar with those which devolved upon the staff placed under her immediate control. Her selection of sisters was most judicious, and this coveted promotion had a magical effect upon the nursing staff, as it placed a premium upon trustworthiness and efficiency. Mrs. Wardroper next induced the governors to increase the number of nurses, a step which enabled her to shorten their hours of duty, and then she succeeded in securing a better dietary for the whole staff. Up to this time it had been almost impossible to organise a proper system of training for the nurses, but this step was effectually taken, when the governors consented to the establishment of the Nightingale Training School in connection with St. Thomas's Hospital in 1860. To the successful development of this school Miss Nightingale devoted all her powers, and it led to the introduction of probationers, who were trained upon a definite system, which attracted educated women, who felt that under the altered conditions nursing offered them a sphere of work not only congenial, but well calculated to promote their own happiness and material welfare. Mrs. Wardroper, with characteristic energy, in order to enable her to better fulfil the duties attaching to the responsible head of a nurse training school, with the invaluable aid of Mr. R. G. Whitfield, the resident medical officer, made it her first business to learn thoroughly the duties of a nurse. Mrs. Wardroper was thus one of the first trained nurses of the Nightingale School, and this fact placed her in a position to understand and enforce discipline and obedience to the orders of the medical men.

### The History of the Nightingale School.

The success and influence of this school for good were largely due to Mrs. Wardroper, and a brief history of its development during the twenty-seven years when she held the office of its superintendent is practically a record of this devoted lady's official life. The school was opened at the old St. Thomas's Hospital in Southwark in June, 1860, with fifteen probationer nurses. The old hospital was purchased by the South-Eastern Railway, two years later, when the patients were removed to a music hall situated in the Surrey Gardens, and its adaptation to the requirements of a hospital, coupled with the provision of accommodation for the nurses and staff in temporary quarters, involved an amount of labour, anxiety, and trouble upon the matron, which the majority of our readers will he able to appreciate from their own experience. The change was made, under these difficult circumstances, with so much success that the governors and medical staff were greatly gratified when the whole of the arrangements were completed. The work of the hospital was carried on in the Surrey Music Hall until September, 1871, when the present St. Thomas's Hospital, on the Albert Embankment, was completed and occupied. During the nine years (1862 to 1871), the difficulties of administration, both in regard to the nursing school and the hospital, were continuous and great. Notwithstanding this, Mrs. Wardroper's administration proved so satisfactory that the Nurses' Training School steadily developed, and grew both in numbers and in public favour. When the new buildings were occupied, it became necessary to increase the number of probationer nurses from 20 to 32—the present number engaged. This school, the first established in this country, and practically the first nurse-training school connected with any large hospital in any country, proved an object lesson, fruitful in results for good, in regard to all the medical institutions throughout the world.

When the Nightingale School was first established, a rule was made that, as vacancies occurred on the staff of St. Thomas's, they should be filled up from the nurses trained in the school until the whole staff had been so trained, and this wise practice has continued up to the present day. A few gentlewomen, admitted to training when the system of probationers was first introduced, soon qualified themselves as ward sisters at St. Thomas's, and were much sought for to fill higher appointments in other hospitals. Many applications were made, not only for matrons, but for nurses, and, after some experiments, the plan of sending nurses singly to other hospitals and infirmaries was given up in favour of one by which the authorities of the Nightingale School sent the whole or a considerable portion of the nursing staff under a superintendent to take charge of other hospitals. The progress of this movement will be readily understood from a few examples :—

In 1862 four nurses were sent to the Royal Infirmary at Liverpool in connection with which a training school for nurses had recently been established by the lady superintendent, Miss Merryweather, who had passed through a short period of training at St. Thomas's. This experiment proved so successful that it led to the establishment of an organisation for nursing the sick poor at their own homes throughout Liverpool. It is interesting to note that the superintendent at the Liverpool Infirmary, Miss Stains, who has but recently passed away, was a former probationer, and that she was one of the ablest and most capable lady superintendents of the present day.

In 1866, the Derbyshire General Infirmary was taken over by a matron supplied by the Nightingale School. In 1872, Miss Barclay, with a staff of 13 nurses, was sent to the Royal Infirmary at Edinburgh to take charge of a portion only of the old hospital, the condition of which was, as we stated in a recent article, so bad at that time as to be remarkable.

In 1877, Miss Williams was appointed Matron of St. Mary's Hospital, Paddington. Several ward sisters and nurses were supplied to her, and subsequently a training school was established there, which has proved most successful and popular. In 1878, Miss Machin was appointed Matron of St. Bartholomew's Hospital. In the course of the same year Mrs. Linnick was appointed Matron of the Dublin Nurses' Training Institution, and of Sir Patrick Dun's Hospital.

In 1880, Miss Pine was appointed Matron of the Westminster Hospital and Superintendent of its training school and home, founded by Lady Augusta Stanley. It would take too long to give the names of all the hospitals in the country, which have been supplied with matrons, or superintendents, or nurses, or all three, from the Nightingale Training School. The instances we have given prove to demonstration the enormous influence for good which the Nightingale School has exercised upon nursing in public institutions throughout Great Britain and Ireland, a fact which we make bold to say, has not been adequately recognized by the public, who, probably for want of precise knowledge, have heretofore failed to recognize that practically the first dawn of a better state of things in regard to nursing the sick has everywhere been due to the teachings of Miss Nightingale, as enforced and developed by Mrs. Wardroper.

Mrs. Wardroper's influence was not confined, however, to nursing in public institutions in this country. So long ago as December, 1867, in consequence of an application from the Government of New South Wales, Miss Osborne was appointed Lady Superintendent of the Sydney Infirmary, with a staff of five nurses, and in 1875 Miss Machin, accompanied by four head-nurses, took up the post of Matron of the General Hospital at Montreal, Canada. The good work was not, however, confined to the Colonies, for, in 1882, Miss Fuhrmann, who had been sent for training by Her Royal and Imperial Highness, the Crown Princess of Prussia, took charge of the Nurses' Home, established by the Crown Princess in Berlin, and subsequently became Superintendent of nurses at the Berlin City Hospital.

Liverpool was so well satisfied with the improvements at the Royal Infirmary, already referred to, that, three years later, the Liverpool Select Vestry invited Miss Agnes Jones, a name that will always be honoured in the nursing world, to become Superintendent of the great Liverpool Workhouse Infirmary, and to provide it with a staff of nurses. The infirmary contained from 1,000 to 1,500 beds, and it was the first to introduce trained, in lieu of pauper nurses, with a result so eminently successful that a training school for nurses was subsequently attached to the infirmary, which has been largely instrumental in extending the benefits of trained nurses, especially in workhouse infirmaries. In 1881, Miss E. Vincent left the Lincoln County Hospital to become Matron of the Marylebone New Workhouse Infirmary with 700 beds. She was accompanied by 13 nurses, and soon established a training school for infirmary nurses, which is one of the most successful in the country. In 1885, Miss Styring became Matron of the Paddington New Workhouse Infirmary; she was accompanied by five head nurses, and has made this institution one of the most efficient in London.

In 1866 the Manchester Nurses' Training Institution was supplied with a staff of nurses, and in 1875. when that most able administratcr, Mrs. Dacre Craven (then Miss Florence Lees), organised the Metropolitan and National Nursing Association, for providing trained nurses for visiting the sick poor at their own homes, she selected a staff of nurses from the class of special or lady probationers, and a large number of trained nurses have since been supplied to this society and its various branches. This step emphasised and brought home to the public the importance of trained nursing, by extending its benefits beyond the immediate sphere of hospital work, and supplying the poor with the attendance of skilled nurses at their own homes. It is surprising, and gratifying to be able to record that in 1869 the fame of the Nightingale School had so grown, that Miss Deeble was appointed by the War Office Superintendent of Nurses at the Royal Victoria Hospital, Netley, whither she was accompanied by a staff of six nurses to act as ward sisters. It may further be noted that in 1882 five nurses, and in 1884 three nurses from the Nightingale School went to serve with the army in Egypt.

It will thus be seen that the first seeds of improvement in nursing have practically everywhere been sown by the Nightingale School, of which Mrs. Wardroper was for 27 years the executive chief. We say "everywhere," because it was a Nightingale nurse who first introduced trained nurses to Philadelphia in the United States, and although the late Dr. Whittemore had done much in the same direction at the Massachusetts General Hospital at Boston, and the Belle Vue Nurse-Training School at New York has long flourished and done yeoman's service for nursing in the United States, still each and all would readily admit that originally they owed most, if not all, the cardinal points of their system to the Nightingale School. It is pleasant in this connection to quote the following from the report of the Nightingale Fund for the year 1886, to which we are indebted for many of the facts mentioned: "St. Thomas' has thus become the mother house of a long list of pupils, who looked up to Mrs. Wardroper for advice and help, which was always willingly given to the best of her ability. The object for which the school was established has been attained gradually, quietly, without ostentation, and with perfect harmony on all sides, and this success has been largely due to the management of Mrs. Wardroper. It must, the Committee think, be acknowledged that the present improved condition in the nursing of hospitals generally, whether in any way connected with the school or not, whether having regard to their internal administration or to the many good training schools for nurses which have been established in connection with them, has owed much to St. Thomas's Hospital, to the Nightingale Fund School, and to Mrs. Wardroper as the Matron of the hospital and Superintendent of the school."

At the annual meeting of the Nightingale Fund, held April 25th, 1887, Mr. Rathbone, M.P., speaking on behalf of the council, expressed a deep sense of the inestimable services which Mrs. Wardroper had rendered to the Nightingale Fund School, as well as to the nursing profession generally, and stated the conviction that to her great administrative ability, and unsparing and sympathetic devotion, were chiefly due—under the counsels of Miss Nightingale—the success of the school, and the acknowledged influence which it had exercised.

### Proposed Memorial.

Mrs. Wardroper only lived to enjoy her well-earned retirement for a little over five years; they were unfortunately attended by much sorrow, suffering, and infirmity. America wisely recognised the services rendered to nursing by Miss Fisher in Philadelphia, short though they were, and we feel moved to express a hope that steps will be taken to commemorate the memory and the work of the mother of trained nursing in England by the institution without delay of some suitable and adequate memorial. We knew Mrs. Wardroper during the greater part of the time she was superintendent of the Nightingale School; we had many opportunities of realising her character and the quality of her work; we always found her ready to give advice, counsel, or help, and the amount of time which she spent in writing, so that those interested in nursing who resided far away might have the benefit of her knowledge and experience must have deprived her of much well deserved and necessary leisure. Mrs. Wardroper was a great woman: great in practical usefulness, in untiring devotion to duty, in securing immensely successful results, and in the possession of a humility which caused her to seek retirement and to keep her name in the background when justice demanded that she should have full public recognition for services so pre-eminently valuable as to place them beyond the power of all human recompense. We feel confident that there must be a very large number of people who will share our feelings, and desire to co-operate in raising a suitable memorial, so that the life-work of this grand woman may live and fructify, and may remain on record for all time as an example and encouragement to women workers all the world over. We shall be glad to receive communications on the subject without delay.

## Figures and Tables

**Figure f1:**